# Supplementary material for: PRMT2 promotes HIV-1 latency by preventing nucleolar exit and phase separation of Tat into the Super Elongation Complex
Source: Nat Commun. 2023 Nov 10;14:7274. doi: 10.1038/s41467-023-43060-1 (PMC10638354; doi:10.1038/s41467-023-43060-1)
Supplement: Supplementary file 1 — Supplementary information [file 41467_2023_43060_MOESM1_ESM.pdf]

Supplementary information inventory

PRMT2 promotes HIV-1 latency through preventing nucleolar exit and phase separation of Tat into the Super Elongation Complex

Jiaxing Jin[1#], Hui Bai[1#], Han Yan[1], Ting Deng[2], Tianyu Li[3], Ruijing Xiao[3], Lina Fan[4], Xue Bai[5], Hanhan Ning[1], Zhe Liu[5], Kai Zhang[5], Xudong Wu[5], Kaiwei Liang[3], Ping Ma[4*], Xin Gao[6*], Deqing Hu[1*]

Correspondence to: Deqing Hu (hudq@tmu.edu.cn), Xin Gao (gaoxin1@ihcams.ac.cn) or Ping Ma (docmaping@outlook.com).

This PDF file includes:

Supplementary figures 1 to 7

Figure S1

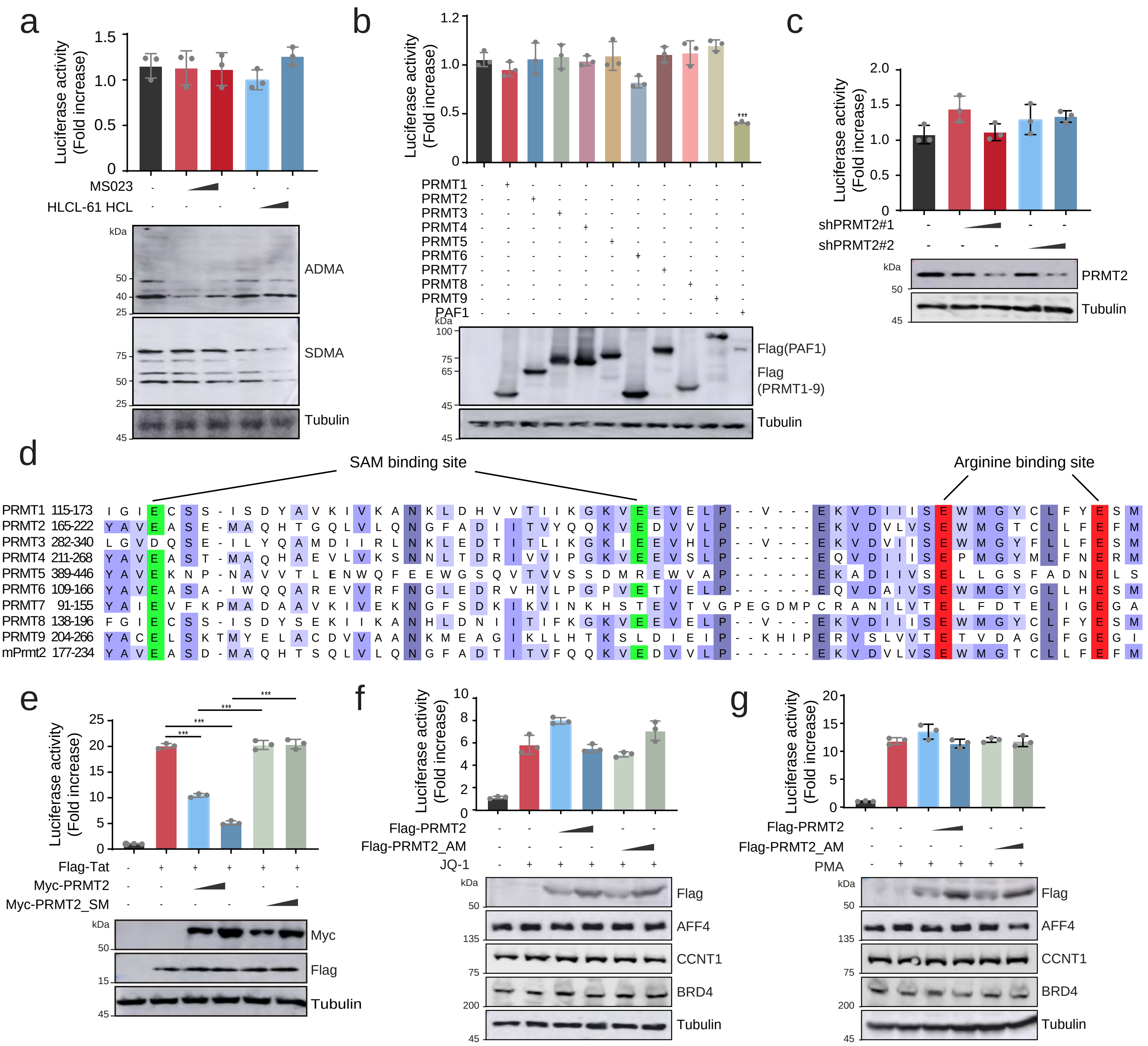

a
Luciferase activity (Fold increase)
MS023
HLCL-61 HCL
kDa
ADMA
SDMA
Tubulin
b
Luciferase activity (Fold increase)
PRMT1
PRMT2
PRMT3
PRMT4
PRMT5
PRMT6
PRMT7
PRMT8
PRMT9
PAF1
Flag(PAF1)
Flag (PRMT1-9)
Tubulin
c
Luciferase activity (Fold increase)
shPRMT2#1
shPRMT2#2
PRMT2
Tubulin
d
SAM binding site
Arginine binding site
PRMT1 115-173
PRMT2 165-222
PRMT3 282-340
PRMT4 211-268
PRMT5 389-446
PRMT6 109-166
PRMT7 91-155
PRMT8 138-196
PRMT9 204-266
mPrmt2 177-234
e
Luciferase activity (Fold increase)
Flag-Tat
Myc-PRMT2
Myc-PRMT2_SM
Myc
Flag
Tubulin
f
Luciferase activity (Fold increase)
Flag-PRMT2
Flag-PRMT2_AM
JQ-1
Flag
AFF4
CCNT1
BRD4
Tubulin
g
Luciferase activity (Fold increase)
Flag-PRMT2
Flag-PRMT2_AM
PMA
Flag
AFF4
CCNT1
BRD4
Tubulin

Supplementary fig. 1 PRMT2 does not modulate the basal transcriptional activity of HIV-1 LTR, related to fig. 1.

a Basal HIV-1 LTR activity in 293T cells treated for 6 hours with PRMTs inhibitors.

b Basal HIV-1 LTR activity in 293T cells exogenously expressing indicated PRMT family member for 24 h.

c Basal HIV-1 LTR activity in 293T cells depleted for PRMT2 expression by two lentiviral shRNAs.

d Alignment of amino acid sequences in the catalytic core region across human PRMT family members and mouse Prmt2 by Clustal Omega. Conserved residues mediating the binding to SAM and arginine residue of substrates were shaded in dark green and red backgrounds, respectively.

e Tat-induced HIV-1 LTR activity in 293T cells ectopically expressing wild-type or SAM binding-defective mutant (referred to as SM) PRMT2.

f Basal HIV-1 LTR activity in 293T cells ectopically expressing wild-type PRMT2 or AM mutant followed by JQ-1 stimulation for 24 hours.

g Basal HIV LTR activity in 293T cells transfected with wild-type or AM PRMT2 construct followed by PMA stimulation for 24 hours.

Quantifications in (a-c and e-g) are represented as mean±SD from biological triplicates. All western blots are representative of three independent experiments. Statistical significance (a-c and e-g) was determined using a two-tailed Student’s t-test . *$p < 0.05$; **$p < 0.01$; ***$p < 0.001$.

Figure S2

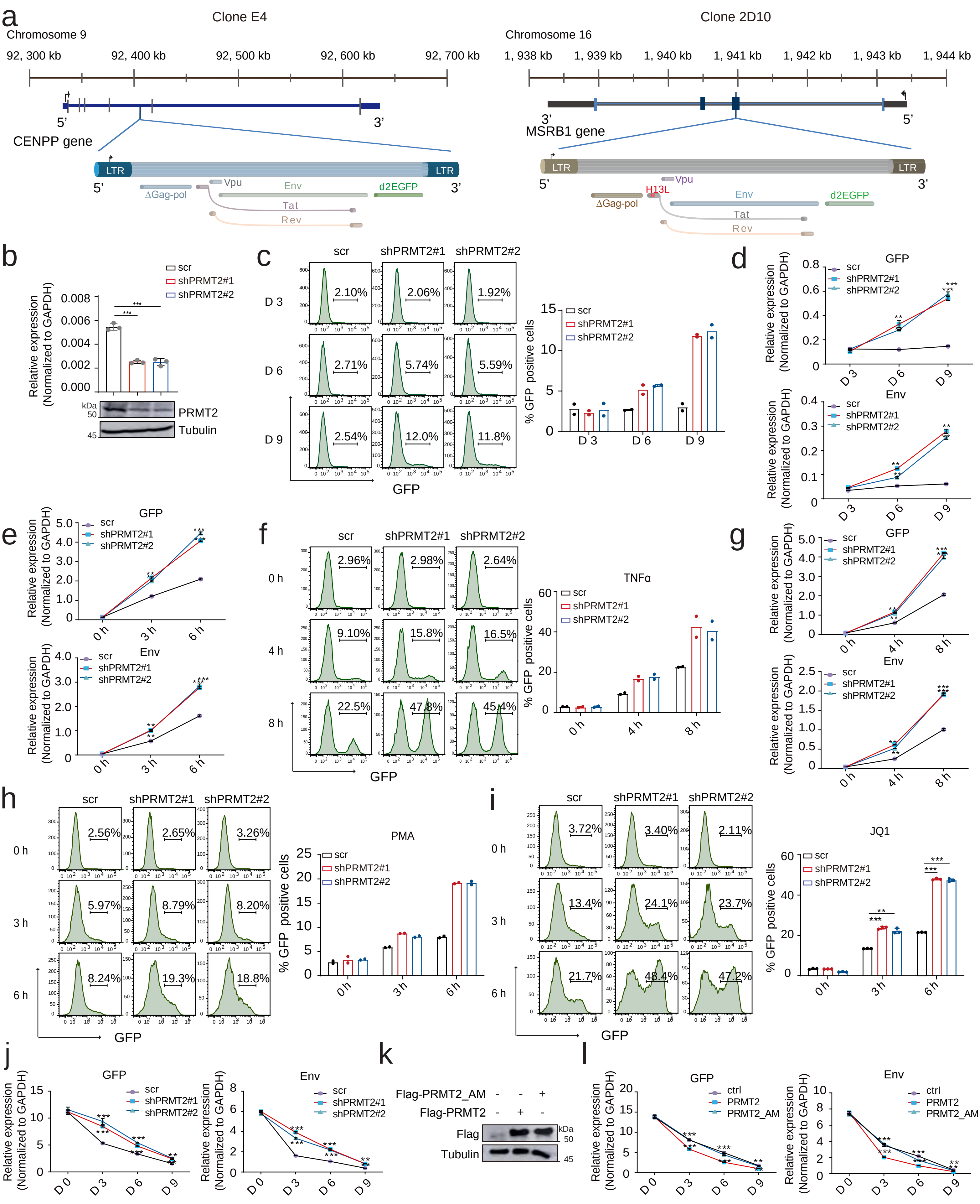
a
Clone E4
Chromosome 9
92, 300 kb
92, 400 kb
92, 500 kb
92, 600 kb
92, 700 kb
CENPP gene
LTR
ΔGag-pol
Vpu
Env
d2EGFP
Tat
Rev
Clone 2D10
Chromosome 16
1, 938 kb
1, 939 kb
1, 940 kb
1, 941 kb
1, 942 kb
1, 943 kb
1, 944 kb
MSRB1 gene
H13L
b
scr
shPRMT2#1
shPRMT2#2
Relative expression (Normalized to GAPDH)
PRMT2
Tubulin
kDa
c
D 3
D 6
D 9
GFP
% GFP positive cells
d
e
f
TNFα
g
h
PMA
i
JQ1
j
k
Flag-PRMT2_AM
Flag-PRMT2
Flag
l
ctrl
PRMT2
PRMT2_AM

Supplementary fig. 2 PRMT2 and its methylase activity are crucial for the suppression of viral transcription to establish and maintain HIV-1 latency, related to fig. 2.

a Schemes of replication-incompetent HIV provirus in Jurkat T-derived E4 and 2D10 clonal cells. HIV-1 provirus in both clones lacks a Gag-pol sequence but contains a d2EGFP in place of the Nef region. E4 contains a wild-type Tat while histidine 13 in Tat was substituted with a leucine residue in the 2D10 clone.

b RT-qPCR and immunoblotting analyses of PRMT2 levels in 2D10 cells 72 hours post lentiviral transduction with two independent shRNAs.

c Representative flow histograms and quantification of GFP^+ cells at indicated days post PRMT2 depletion in 2D10 cells.

d RT-qPCR analyses of GFP (upper panel) and HIV-1 env (lower panel) mRNA levels in 2D10 cells treated as in **b**.

e RT-qPCR analyses of GFP (upper panel) and HIV-1 env (lower panel) mRNA levels in E4 cells stimulated with TNFα for indicated times.

f Representative flow histograms and quantification of GFP^+ cells in control and PRMT2-depleted 2D10 cells stimulated with TNFα for indicated times.

g RT-qPCR analyses of GFP (upper panel) and HIV-1 env (lower panel) mRNA levels in 2D10 cells treated as in (**f**).

h Representative flow histograms and quantifications of GFP^+ cells in control and PRMT2-depleted 2D10 cells stimulated with PMA for indicated times.

i Representative flow histograms and quantificaiton of GFP^+ cells in control and PRMT2-depleted E4 cells stimulated with JQ1 for indicated times.

j RT-qPCR analyses of GFP and env mRNA levels at indicated days following TNFα washout in fully activated control and PRMT2-depleted 2D10 cells by overnight TNFα stimulation.

k Immunoblotting analyses of PRTM2 expression in 2D10 cells as described in fig.2j.

l RT-qPCR analyses of GFP and Env mRNA levels at indicated days following TNFα washout in fully activated control, wild-type, and AM PRMT2-expressing 2D10 cells by overnight TNFα stimulation.

Quantifications in (b, d, e, g, i, j and l) are shown as mean ± SD from biological triplicates. Quantifications in (c, f and h) are shown as mean from biological dublicates Statistical significance (b, d, e, g, i, j and l) was determined using a two-tailed Student’s t-test. $*p < 0.05$; $**p < 0.01$; $***p < 0.001$.

Figure S3

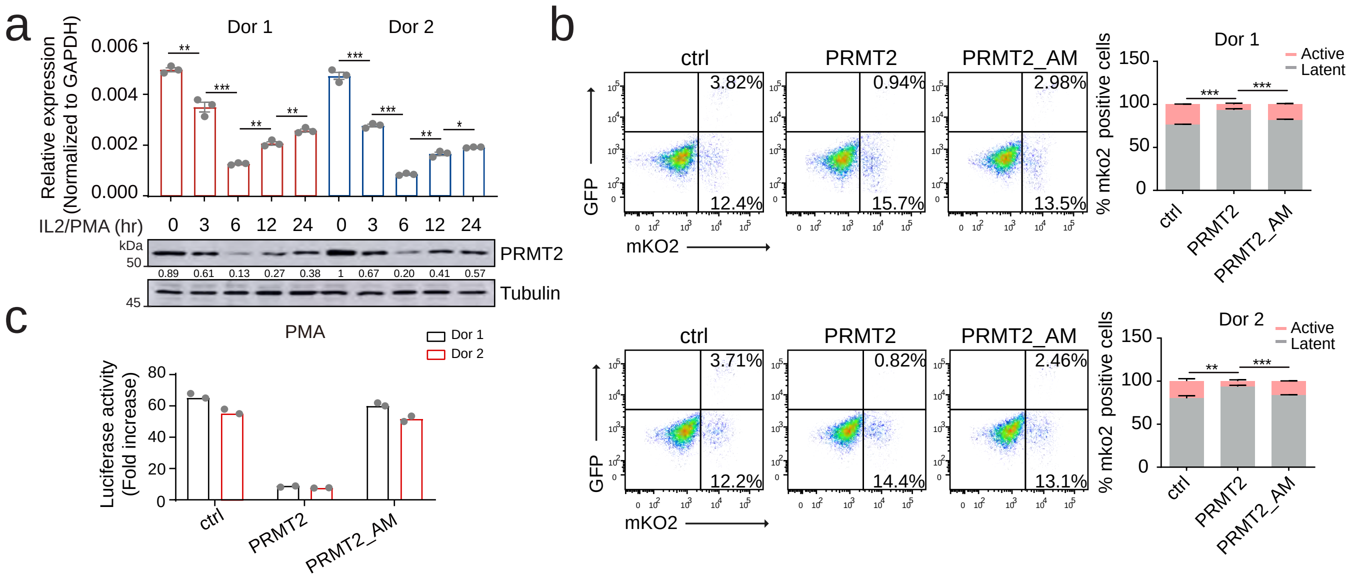
a
Dor 1
Dor 2
Relative expression
(Normalized to GAPDH)
0.006
0.004
0.002
0.000
**
***
**
**
***
***
**
*
IL2/PMA (hr)
0 3 6 12 24 0 3 6 12 24
kDa
50
0.89 0.61 0.13 0.27 0.38 1 0.67 0.20 0.41 0.57
PRMT2
45
Tubulin
b
ctrl
PRMT2
PRMT2_AM
3.82%
12.4%
0.94%
15.7%
2.98%
13.5%
GFP
mKO2
Dor 1
Active
Latent
% mko2 positive cells
150
100
50
0
***
***
ctrl
PRMT2
PRMT2_AM
ctrl
PRMT2
PRMT2_AM
3.71%
12.2%
0.82%
14.4%
2.46%
13.1%
GFP
mKO2
Dor 2
Active
Latent
% mko2 positive cells
150
100
50
0
**
***
ctrl
PRMT2
PRMT2_AM
c
PMA
Dor 1
Dor 2
Luciferase activity
(Fold increase)
80
60
40
20
0
ctrl
PRMT2
PRMT2_AM

Supplementary fig. 3 PRMT2 represses viral transcription to promote latency establishment and maintenance in primary $CD4^+$ T cells of healthy donors and HIV-infected patients, related to fig. 3.

a RT-qPCR analysis of PRMT2 expression in primary $CD4^+$ T cells from two healthy donors that were either left untreated or activated with IL-2 plus PMA for indicated time points. Quantifications were shown as mean±SD (n=3).

b Primary $CD4^+$ T cells were infected with HIV-1_{GKO} virus following ectopic expression of indicated PRMT2 as outlined in fig. 3f. Productively and latently infected cells of a representative experiment were shown in flow pseudocolor dot plots and the ratio of respective cells relative to total infected cells was represented as means ±SD (n=3).

c Primary $CD4^+$ T cells were treated as in fig. 3h. Luciferase activity of an equal number of cells was shown as means (n=2).

Statistical significance (a and b) was determined using a two-tailed Student's t-test. *$p < 0.05$; **$p < 0.01$; ***$p < 0.001$.

Figure S4

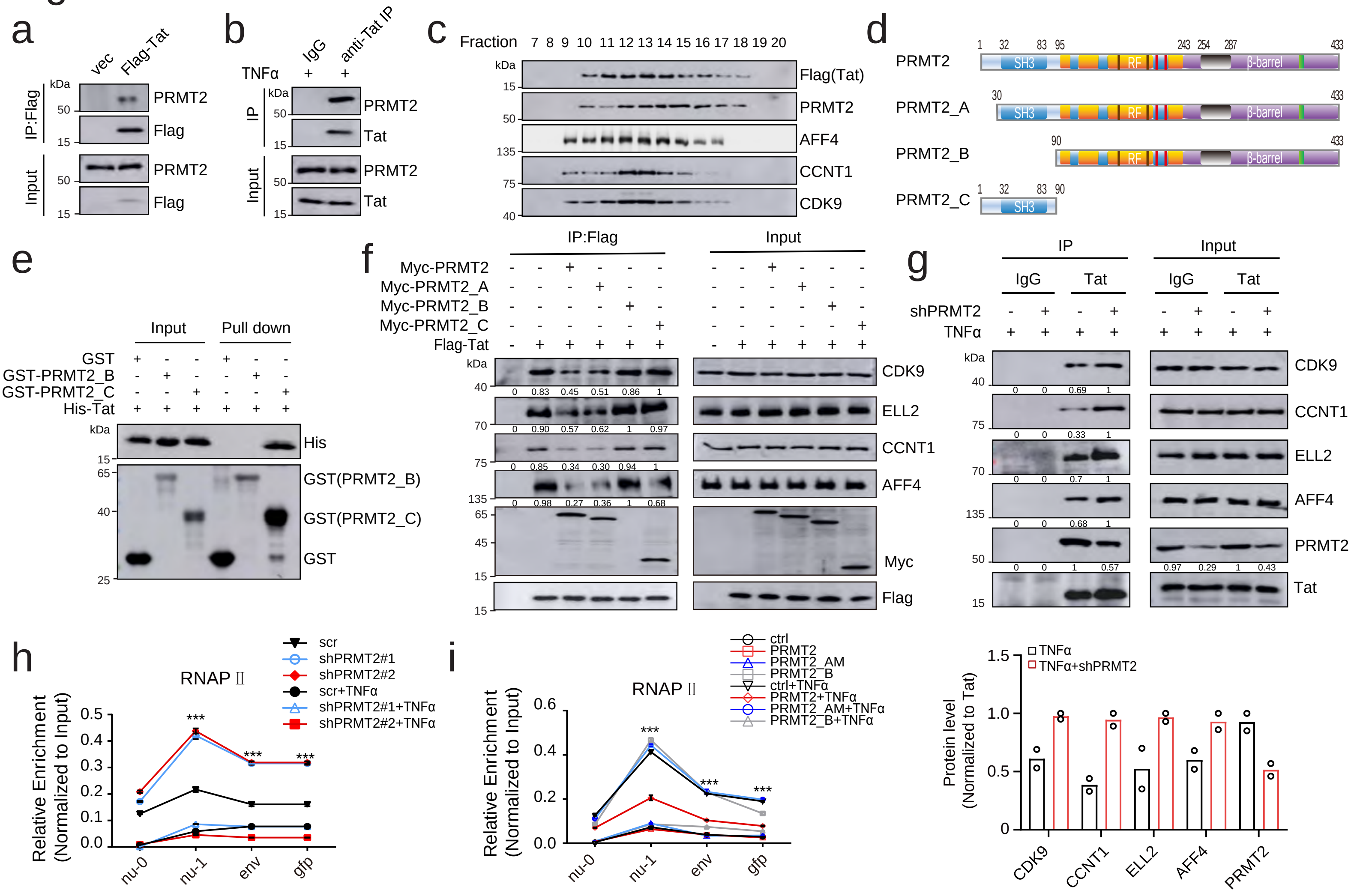

a
vec
Flag-Tat
IP:Flag
Input
PRMT2
Flag
b
IgG
anti-Tat IP
TNFα
IP
Input
PRMT2
Tat
c
Fraction 7 8 9 10 11 12 13 14 15 16 17 18 19 20
Flag(Tat)
PRMT2
AFF4
CCNT1
CDK9
d
PRMT2
PRMT2_A
PRMT2_B
PRMT2_C
SH3
RF
β-barrel
e
Input
Pull down
GST
GST-PRMT2_B
GST-PRMT2_C
His-Tat
His
GST(PRMT2_B)
GST(PRMT2_C)
GST
f
IP:Flag
Input
Myc-PRMT2
Myc-PRMT2_A
Myc-PRMT2_B
Myc-PRMT2_C
Flag-Tat
CDK9
ELL2
CCNT1
AFF4
Myc
Flag
g
IP
Input
IgG
Tat
shPRMT2
TNFα
CDK9
CCNT1
ELL2
AFF4
PRMT2
Tat
h
RNAP Ⅱ
Relative Enrichment (Normalized to Input)
scr
shPRMT2#1
shPRMT2#2
scr+TNFα
shPRMT2#1+TNFα
shPRMT2#2+TNFα
nu-0
nu-1
env
gfp
i
RNAP Ⅱ
ctrl
PRMT2
PRMT2_AM
PRMT2_B
ctrl+TNFα
PRMT2+TNFα
PRMT2_AM+TNFα
PRMT2_B+TNFα
TNFα
TNFα+shPRMT2
Protein level (Normalized to Tat)
CDK9
CCNT1
ELL2
AFF4
PRMT2

Supplementary fig. 4 PRTM2 binds to Tat and decreases the cellular abundance of the Tat-SEC complex,related to fig. 4.

a Immunoblotting analyses of the nuclear extract and anti-flag immunoprecipitates from 293T cells stably expressing an empty vector or flag-tagged Tat.

b Immunoblotting analyses of the nuclear extract, anti-IgG, and anti-Tat immunoprecipitates from E4 cells stimulated with TNFα for 6 hours.

c Immunoblotting analyses of the fractionated samples from glycerol density gradient ultracentrifugation using the nuclear extracts of 293T cells stably transduced with flag-Tat.

d Schematic representation of domain organization for full-length and the various truncating PRMT2 mutants.

e Purified his-tagged Tat were incubated with immobilized GST or indicated GST-fused PRMT2 variant and then subjected to immunoblotting analyses.

f Immunoblotting analyses of nuclear extract and anti-flag immunoprecipitates from 293T cells co-transfected with vectors expressing flag-Tat and myc-tagged full-length PRMT2 or truncating variants.

g E4 cells with or without endogenous PRMT2 depletion were treated with TNFα overnight and the nuclear extracts were subjected to anti-IgG and anti-Tat immunoprecipitation, followed by immunoblotting analyses with indicated antibodies.Quantification was shown as mean (n=2).

h ChIP-qPCR analyses of RNA Pol II distribution across the proviral genome in E4 cells depleted for endogenous PRMT2 and stimulated with TNFα for 6 hours. Quantification was normalized to input and shown as mean ± SD from biological triplicates.

i ChIP-qPCR analyses of RNA Pol II distribution across the proviral genome in E4 cells transduced with indicated PRMT2 expression construct and stimulated with TNFα for 6 hours. Quantification was normalized to input and shown as mean ± SD from biological triplicates.

Values from densitometric analyses of immunoblots are shown beneath the respective gel bands. Statistical significance (h and i) was determined using a two-tailed Student's t-test. $*p < 0.05$; $**p < 0.01$; $***p < 0.001$.

Figure S5

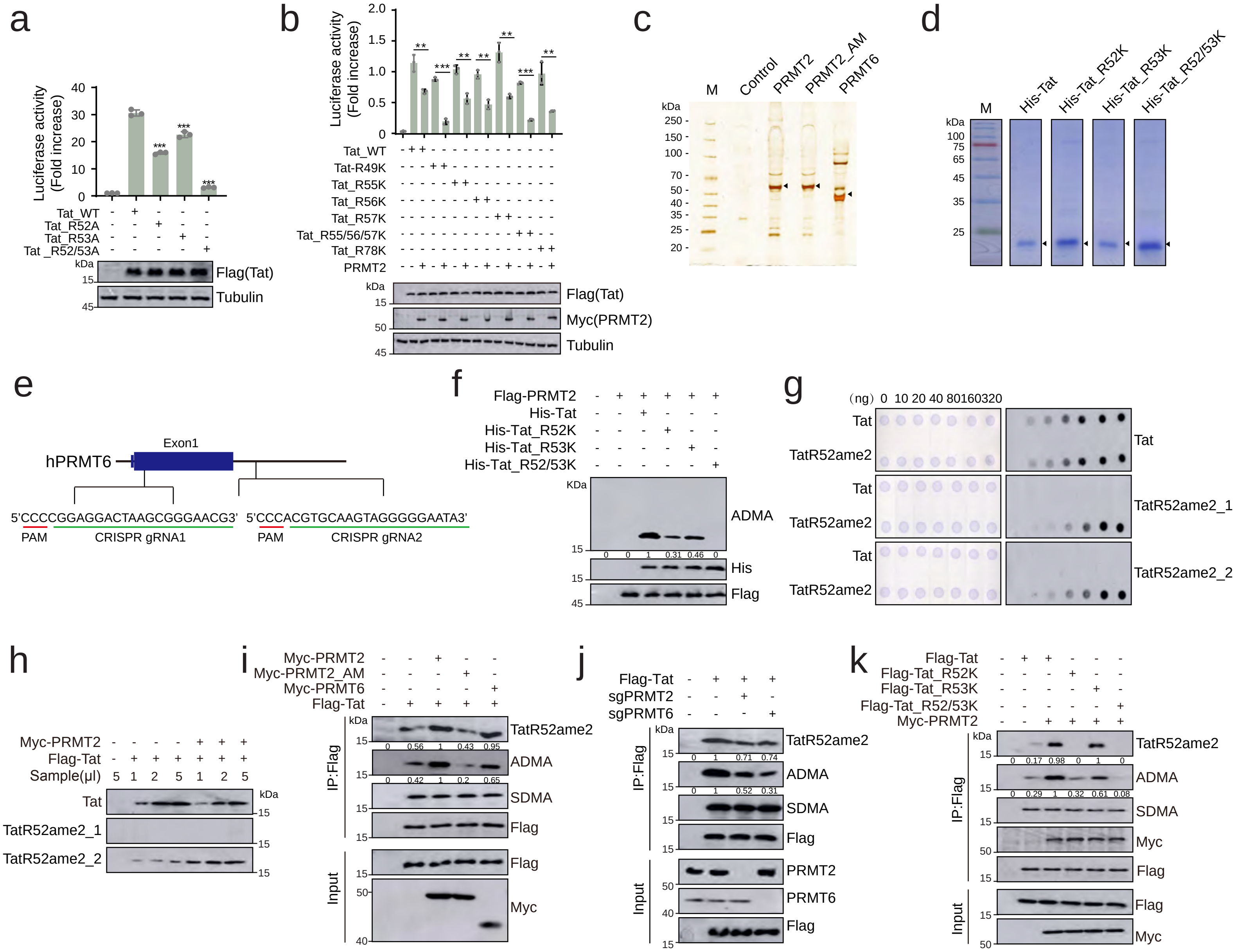

a
b
c
d
e
f
g
h
i
j
k
Luciferase activity (Fold increase)
Tat_WT
Tat_R52A
Tat_R53A
Tat _R52/53A
Flag(Tat)
Tubulin
Tat-R49K
Tat_R55K
Tat_R56K
Tat_R57K
Tat_R55/56/57K
Tat_R78K
PRMT2
Myc(PRMT2)
M
Control
PRMT2
PRMT2_AM
PRMT6
His-Tat
His-Tat_R52K
His-Tat_R53K
His-Tat_R52/53K
hPRMT6
Exon1
5'CCCCGGAGGACTAAGCGGGAACG3'
5'CCCACGTGCAAGTAGGGGGAATA3'
PAM
CRISPR gRNA1
CRISPR gRNA2
Flag-PRMT2
ADMA
His
Flag
Tat
TatR52ame2
TatR52ame2_1
TatR52ame2_2
Myc-PRMT2
Flag-Tat
Sample(μl)
Myc-PRMT2_AM
Myc-PRMT6
IP:Flag
Input
SDMA
Myc
sgPRMT2
sgPRMT6
PRMT6
Flag-Tat_R52K
Flag-Tat_R53K
Flag-Tat_R52/53K

Supplementary fig. 5 PRMT2 methylates R52 of Tat to attenuate its transactivating activity, related to Figure 5.

a HIV-1 LTR luciferase activity in 293T cells ectopically expressing wild-type or the indicated arginine-to-alanine mutant Tat was represented as mean±SD from biological triplicates.

b HIV-1 LTR luciferase activity was represented as mean±SD from biological triplicates in 293T cells ectopically expressing wild-type or indicated arginine-to-lysine mutant Tat alone, or together with co-expression of exogenous PRMT2.

c Silver staining visualization of anti-flag immuno-affinity eluates from the nuclear extracts of 293T cells stably expressing either flag-tagged wild-type, its methylase-inactive mutant, and PRMT6. Arrowheads indicate the flag-tagged protein.

d Coomassie blue staining of his-tagged wild-type and the indicated recombinant mutant Tat expressed and purified from E.coli using affinity chromatography with Ni-NTA agarose.

e Graphical representation of the human *PRMT6* loci and the sequence for CRISPR/Cas9 guide RNAs designed to knock out its expression.

f Immunoblotting analyses of *in vitro* arginine methylation of indicated his-tagged recombinant Tat protein incubated with immuno-affinity purified PRMT2.

g Dot blot evaluation of the specificity and sensitivity of antibodies raised against unmodified and R52ame2 Tat synthetic peptides.

h Immunoblotting evaluation of the specificity and sensitivity of Tat R52ame2 antibodies using the nuclear extracts of 293T cells expressing flag-Tat alone or in combination with PRMT2.

i Immunoblotting analyses of Tat R52 methylation in 293T cells expressing wild-type PRMT2, mutant PRMT2, and PRMT6.

j Immunoblotting analyses of Tat R52 methylation in 293T cells with depletion of PRMT2 or PRMT6.

k Methylation of wild-type or the indicated mutant Tat in 293T cells with or without PRMT2 co-expression was analyzed by immunoblotting.

Values from densitometric analyses of immunoblots are shown beneath the respective gel bands and statistical significance (a and b) was determined using a two-tailed Student's t-test. $^*p < 0.05$; $^{**}p < 0.01$; $^{***}p < 0.001$.

Figure S6

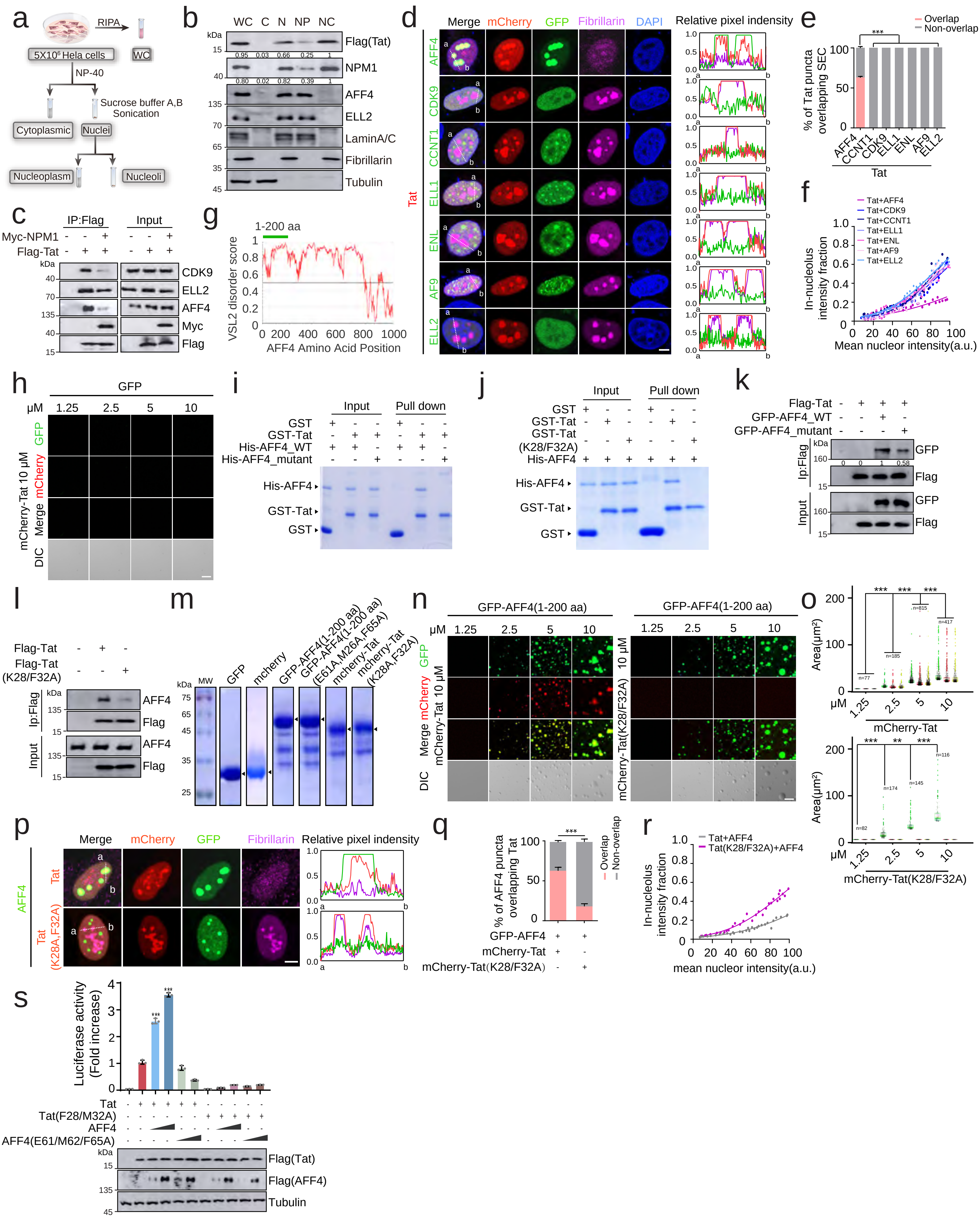

a
RIPA
5X10⁶ Hela cells
WC
NP-40
Sucrose buffer A,B
Sonication
Cytoplasmic
Nuclei
Nucleoplasm
Nucleoli
b
WC C N NP NC
Flag(Tat)
NPM1
AFF4
ELL2
LaminA/C
Fibrillarin
Tubulin
c
IP:Flag
Input
Myc-NPM1
Flag-Tat
CDK9
ELL2
AFF4
Myc
Flag
d
Merge mCherry GFP Fibrillarin DAPI
Relative pixel indensity
Tat
AFF4
CDK9
CCNT1
ELL1
ENL
AF9
ELL2
e
Overlap
Non-overlap
% of Tat puncta overlapping SEC
Tat
f
Tat+AFF4
Tat+CDK9
Tat+CCNT1
Tat+ELL1
Tat+ENL
Tat+AF9
Tat+ELL2
In-nucleolus intensity fraction
Mean nucleor intensity(a.u.)
g
1-200 aa
VSL2 disorder score
AFF4 Amino Acid Position
h
GFP
mCherry-Tat 10 μM
Merge
DIC
i
Input
Pull down
GST
GST-Tat
His-AFF4_WT
His-AFF4_mutant
His-AFF4
GST-Tat
GST
j
Input
Pull down
GST
GST-Tat
GST-Tat (K28/F32A)
His-AFF4
k
Flag-Tat
GFP-AFF4_WT
GFP-AFF4_mutant
Ip:Flag
Input
GFP
Flag
l
Flag-Tat
Flag-Tat (K28/F32A)
Ip:Flag
Input
AFF4
Flag
m
MW
GFP
mcherry
GFP-AFF4(1-200 aa)
GFP-AFF4(1-200 aa) (E61A,M26A,F65A)
mcherry-Tat
mcherry-Tat (K28A,F32A)
n
GFP-AFF4(1-200 aa)
mCherry-Tat 10 μM
mCherry-Tat(K28/F32A) 10 μM
Merge
DIC
o
Area(μm²)
mCherry-Tat
mCherry-Tat(K28/F32A)
p
Merge mCherry GFP Fibrillarin
Relative pixel indensity
AFF4
Tat
Tat (K28A,F32A)
q
% of AFF4 puncta overlapping Tat
Overlap
Non-overlap
GFP-AFF4
mCherry-Tat
mCherry-Tat(K28/F32A)
r
Tat+AFF4
Tat(K28/F32A)+AFF4
In-nucleolus intensity fraction
mean nucleor intensity(a.u.)
s
Luciferase activity (Fold increase)
Tat
Tat(F28/M32A)
AFF4
AFF4(E61/M62/F65A)
Flag(Tat)
Flag(AFF4)
Tubulin

Supplementary fig. 6 AFF4 phase separation recruits Tat into the SEC droplets in the nucleoplasm, related to Figure 6.

a Experimental scheme for subcellular fractionation.

b Immunoblotting analyses of the indicated subcellular fractions of Hela cells transfected with flag-Tat construct.

c Immunoblotting analyses of the nuclear extracts and anti-flag immunoprecipitated proteins in 293T cells transfected with indicated expression constructs.

d Images (left) and line scan profiles (righ) of Hela cells transfected with indicated constructs.

e Bar plot showing Tat puncta overlapping with GFP dots in (**d**) (n=20 fields) .

f Fraction of in-nucleolar Tat puncta fluorescence intensity as a function of mean nuclear intensity in Hela cells in (**d**). Each dot represents one cell (n=30).

g Intrinsic disorder regions across full-length AFF4 were predicted by PONDR (Predictor of Natural Disordered Regions), VSL2 algorithm.

h Confocal images of *in vitro* droplet formation in a solution containing indicated recombinant proteins.

i, j GST pull down assay showing interaction of Tat with wild-type and mutant AFF4 (i) or AFF4 with wild-type and mutant Tat (j).

k, l Immunoblotting analyses the anti-flag immunoprecipitated proteins and the nuclear extracts from 293T cells co-transfected with indicated constructs.

m Coommassie blue staining of the indicated his-tagged recombinant protein.

n Confocal microscopic images of *in vitro* droplet formation in solution containing indicated recombinant proteins.

o Quantification of the size of droplets in (**n**). Center line, median; box, upper and lower quantiles; whisker, maximal and minimal values.

p Images (left) and line scan profiles (right) of Hela cells co-expressing indicated proteins.

q Bar plot showing AFF4 puncta overlapping with Tat dots shown in (**p**) (n=20 fields).

r Fraction of in-nucleolar Tat puncta fluorescence intensity as a function of mean nuclear intensity in Hela cells in (**p**). Each dot represents one cell (n=30).

s HIV-1 LTR-driven luciferase activity in 293T cells 36 hours post transfection with indicated constructs were shown as mean±S.D (n=3).

Scale bars in (d and p, 5 μm) and in (h and n, 10 μm). Values from densitometric analyses of immunoblots are shown beneath the respective gel bands and statistical significance was determined using a two-tailed Student's t-test (e, q and s) and two-sided Wilcoxon test (o) $*p < 0.05$; $**p < 0.01$; $***p < 0.001$.

Figure S7

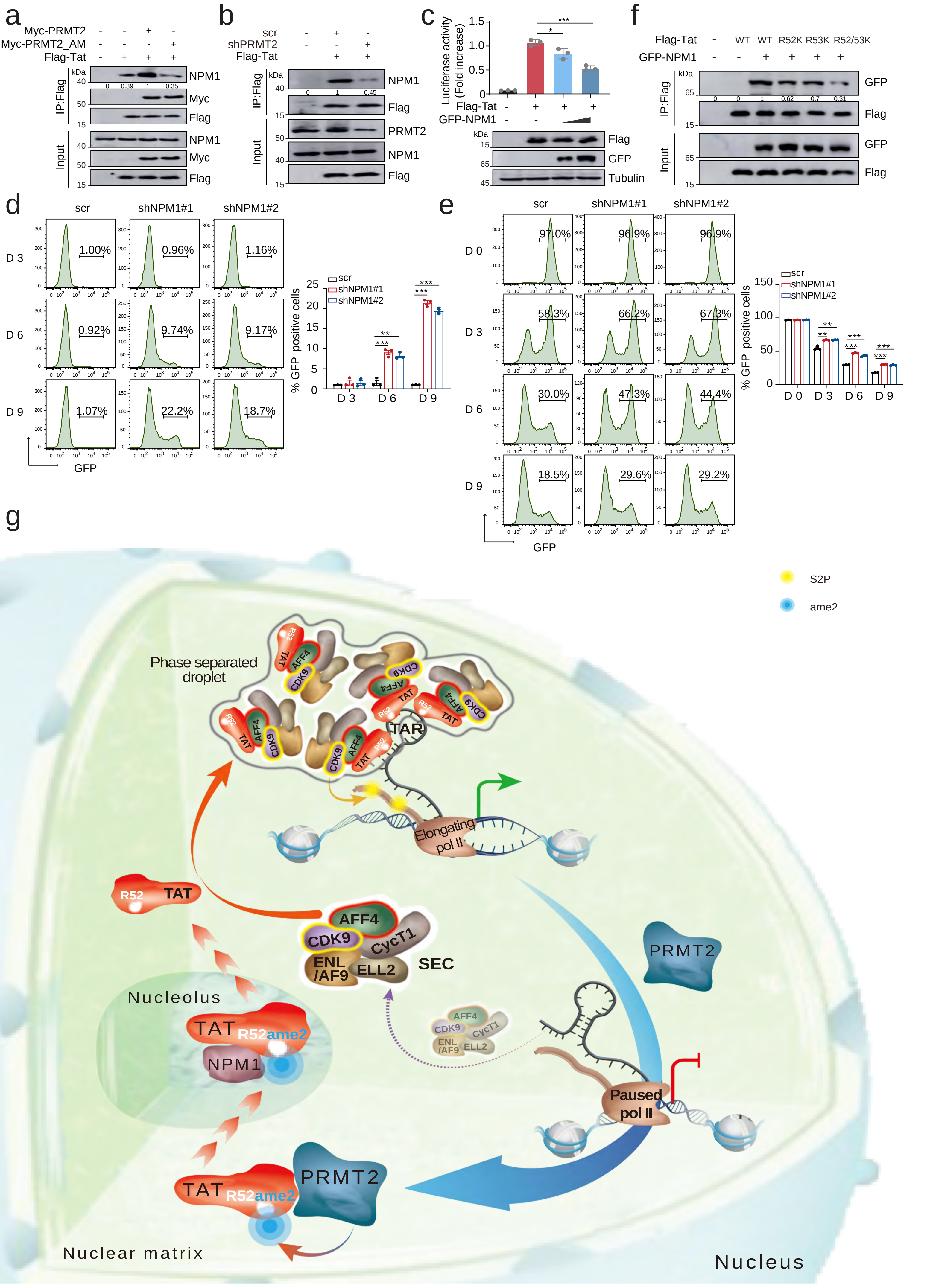

a
Myc-PRMT2
Myc-PRMT2_AM
Flag-Tat
IP:Flag
Input
NPM1
Myc
Flag
0 0.39 1 0.35
b
scr
shPRMT2
Flag-Tat
NPM1
Flag
PRMT2
0 1 0.45
c
Luciferase activity (Fold increase)
Flag-Tat
GFP-NPM1
Flag
GFP
Tubulin
f
Flag-Tat
WT WT R52K R53K R52/53K
GFP-NPM1
GFP
Flag
0 0 1 0.62 0.7 0.31
d
scr
shNPM1#1
shNPM1#2
D 3
D 6
D 9
1.00%
0.96%
1.16%
0.92%
9.74%
9.17%
1.07%
22.2%
18.7%
GFP
% GFP positive cells
e
D 0
97.0%
96.9%
96.9%
58.3%
66.2%
67.3%
30.0%
47.3%
44.4%
18.5%
29.6%
29.2%
g
S2P
ame2
Phase separated droplet
TAR
Elongating pol II
R52
TAT
AFF4
CDK9
CycT1
ENL /AF9
ELL2
SEC
Nucleolus
R52ame2
NPM1
PRMT2
Paused pol II
Nuclear matrix
Nucleus

Supplementary fig. 7 PRMT2 promotes nucleolar sequestration of Tat to attenuate heterotypic Tat-SEC droplet formation, related to Figure 7.

a, b Immunoblotting analyses of the nuclear extract and anti-flag immunoprecipitated proteins from 293T cells co-transfected with indicated ectopic expression (a) or knockdown (b) constructs.

c HIV-1 LTR-driven luciferase activity in 293T cells ectopically expressing indicated proteins was represented as mean ± SD (n=3) .

d Representative flow histograms showing the frequency of GFP^+ population in E4 cells at indicated days post NPM1 depletion. Quantification of the percentage of GFP^+ cells was shown as mean ± SEM (n=3).

e Representative flow histograms showing the frequency of GFP^+ population in 2D10 cells reactivated by overnight treatment of TNFα followed by its washout and continuing culture for indicated times. Quantification was shown as mean ± SEM (n=3) from three biological triplicates.

f Immunoblotting analyses of the nuclear extract and anti-flag immunoprecipitates from 293T cells transfected with indicated Tat expression construct.

g Working model for how Tat function is regulated to mediate the switch between active and latent infection of HIV-1 in $CD4^+$ T cells.

Values from densitometric analyses of immunoblots are shown beneath the respective gel bands and statistical significance (c-e) was determined using a two-tailed Student's t-test. *$p < 0.05$; **$p < 0.01$; ***$p < 0.001$.
