# Supplementary material for: PRMT2 promotes HIV-1 latency by preventing nucleolar exit and phase separation of Tat into the Super Elongation Complex
Source: Nat Commun. 2023 Nov 10;14:7274. doi: 10.1038/s41467-023-43060-1 (PMC10638354; doi:10.1038/s41467-023-43060-1)
Supplement: Supplementary file 3 — Reporting Summary [file 41467_2023_43060_MOESM3_ESM.pdf]

nature portfolio

Corresponding author(s): Deqing Hu

Last updated by author(s): Oct 21, 2023

Reporting Summary

Nature Portfolio wishes to improve the reproducibility of the work that we publish. This form provides structure for consistency and transparency in reporting. For further information on Nature Portfolio policies, see our Editorial Policies and the Editorial Policy Checklist.

Statistics

For all statistical analyses, confirm that the following items are present in the figure legend, table legend, main text, or Methods section.

n/a	Confirmed	
☐	☒	The exact sample size (*n*) for each experimental group/condition, given as a discrete number and unit of measurement
☐	☒	A statement on whether measurements were taken from distinct samples or whether the same sample was measured repeatedly
☐	☒	The statistical test(s) used AND whether they are one- or two-sided<br>*Only common tests should be described solely by name; describe more complex techniques in the Methods section.*
☒	☐	A description of all covariates tested
☒	☐	A description of any assumptions or corrections, such as tests of normality and adjustment for multiple comparisons
☐	☒	A full description of the statistical parameters including central tendency (e.g. means) or other basic estimates (e.g. regression coefficient) AND variation (e.g. standard deviation) or associated estimates of uncertainty (e.g. confidence intervals)
☐	☒	For null hypothesis testing, the test statistic (e.g. *F*, *t*, *r*) with confidence intervals, effect sizes, degrees of freedom and *P* value noted<br>*Give P values as exact values whenever suitable.*
☒	☐	For Bayesian analysis, information on the choice of priors and Markov chain Monte Carlo settings
☒	☐	For hierarchical and complex designs, identification of the appropriate level for tests and full reporting of outcomes
☒	☐	Estimates of effect sizes (e.g. Cohen's *d*, Pearson's *r*), indicating how they were calculated

Our web collection on statistics for biologists contains articles on many of the points above.

Software and code

Policy information about availability of computer code

Data collection	Flow cytometry data was collected on a BD FACSDivaTm. Quantitative PCR data was collected using Bio-Rad CFX Maestro. Fluorescent confocal images were acquired on ZEISS ZEN.
Data analysis	Flow cytometry was analyzed with FlowJo v10.6.2. Statistical analysis data were analyzed with GraphPad software v.9.4. Confocal images and Immunoblotting results were analyzed by ImageJ bundled with 64-bit Java 8.

For manuscripts utilizing custom algorithms or software that are central to the research but not yet described in published literature, software must be made available to editors and reviewers. We strongly encourage code deposition in a community repository (e.g. GitHub). See the Nature Portfolio guidelines for submitting code & software for further information.

Data

Policy information about availability of data

All manuscripts must include a data availability statement. This statement should provide the following information, where applicable:

- Accession codes, unique identifiers, or web links for publicly available datasets
- A description of any restrictions on data availability
- For clinical datasets or third party data, please ensure that the statement adheres to our policy

All original data are provided in Source Data file. GuavaH databaseis accessible via http://www.GuavaH.org.

Research involving human participants, their data, or biological material

Policy information about studies with human participants or human data. See also policy information about sex, gender (identity/presentation), and sexual orientation and race, ethnicity and racism.

Reporting on sex and gender	This study investigated proviral reactivation in HIV-1-infected CD4+ T cells from four individuals on long-term suppressive ART. Sex and gender were not considered as criteria for patient selection because HIV-1 latency and reactivation should not vary very much between male and female patients. Participants were selected based on their well-documented persistent viral suppression with undetectable plasma viremia (<50 copies/ml) and high CD4+T cell number (>500 cells/ml) in peripheral blood for at least two years. All patients provided written informed consent and the study was reviewed and approved by the Ethics Committees of Tianjin Second People's Hospital.
Reporting on race, ethnicity, or other socially relevant groupings	All four patients are Asian chinese. Other socially relevant are not considered in study design.
Population characteristics	The age of four patients are 28, 34, 41 and 41 years old respectively. No genetic characteristics were obtained as a part of the study. Standard ART regimen were administered to these patients.
Recruitment	Gender, age, etc were not considered parameters for selection of participants. Participants were recruited only based on viral load and CD4+T cells in peripheral blood. They were on long-term ART, with plasma viremia less than 50 copies/ml and CD4 +T cell number more than 500 cells/ml. We carried out experiment on in vitro cultured primary cells and thus the conclusions made in our work is unlikely be impacted by participants' gender, age, etc.
Ethics oversight	The Ethics Committees of Tianjin Second People's Hospital approved the use of HIV-1-infected CD4+ T cells for this study.

Note that full information on the approval of the study protocol must also be provided in the manuscript.

Field-specific reporting

Please select the one below that is the best fit for your research. If you are not sure, read the appropriate sections before making your selection.

[x] Life sciences [ ] Behavioural & social sciences [ ] Ecological, evolutionary & environmental sciences

For a reference copy of the document with all sections, see nature.com/documents/nr-reporting-summary-flat.pdf

Life sciences study design

All studies must disclose on these points even when the disclosure is negative.

Sample size	No sample size calclutaion was performed. In most cases, 3 biological triplicates were performed for determining statistical significance.
Data exclusions	No data sets were excluded.
Replication	The majority of experiments were repeated at least three times to ensure reproducibility. All attempts at replication were successful.
Randomization	Randomization is not relevant to this study as no samples/individuals were allocated to experimental groups. Samples and their appropriate controls were<br>processed at the same time.
Blinding	Blinding was not performed in this study. The relevant controls were processed at the same time as the samples and read in an unbiased manner.

Reporting for specific materials, systems and methods

We require information from authors about some types of materials, experimental systems and methods used in many studies. Here, indicate whether each material, system or method listed is relevant to your study. If you are not sure if a list item applies to your research, read the appropriate section before selecting a response.

Materials & experimental systems

n/a	Involved in the study	
	☒	Antibodies
	☒	Eukaryotic cell lines
☒		Palaeontology and archaeology
☒		Animals and other organisms
☒		Clinical data
☒		Dual use research of concern
☒		Plants

Methods

n/a	Involved in the study	
☒		ChIP-seq
	☒	Flow cytometry
☒		MRI-based neuroimaging

Antibodies

Antibodies used

Mouse monoclonal anti-Flag sigma F3165 Dilute at 1:5k;
Rabbit monoclonal anti-beta Tubulin Santa Cruz sc-53140 Dilute at 1:5k;
Mouse monoclonal anti-PRMT6 Santa Cruz sc-271744 Dilute at 1:1k;
Rabbit polyclonal anti-PRMT2 ABclonal A5835 Dilute at 1:5k;
Rabbit monoclonal anti-SDMe-Arginine CST 13222S Dilute at 1:1k;
Rabbit monoclonal anti-ADMe-Arginine CST 13522S Dilute at 1:1k;
Rabbit monoclonal anti-Rpb1 NTD (D8L4Y) CST 14958S Dilute at 1:1k;
Rabbit monoclonal anti-Phospho-Rpb1 CTD (Ser2) (E1Z3G) CST 13499S Dilute at 1:1k;
Rabbit monoclonall anti-Cyclin T1 (D1B6G) CST 81464S Dilute at 1:1k;
Rabbit polyclonal anti-MCEF Bethyl A302-538A Dilute at 1:2k;
Rabbit polyclonal anti-ELL2 Bethyl A302-505A Dilute at 1:2k;
Mouse monoclonal anti-His Santa Cruzsc-14958S Dilute at 1:5k;
Rabbit polyclonal anti-GST CST 2622S Dilute at 1:1k;
Rabbit monocloanl anti-CDK9 CST 2316S Dilute at 1:2k;
Rabbit monoclonal anti-BRD4 CST 13440S Dilute at 1:2k;
Rabbit polyclonal anti-LaminA/C CST 2032S Dilute at 1:2k;
Rabbit monocloncal anti-GFP CST 2956S Dilute at 1:5k;
Rabbit monocloncal anti-Myc CST 2278S Dilute at 1:5k;
Anti-mouse IgG, HRP-linked Antibody CST 7076S Dilute at 1:3k;
Anti-rabbit IgG, HRP-linked Antibody CST 7074S Dilute at 1:3k;
Mouse monoclonal anti-Fibrillarin[38F3] abcam ab4566 Dilute at 1:1k;
Goat anti-Mouse IgG (H+L) Highly Cross-Adsorbed Secondary Antibody, Alexa Fluor™ Plus 647 Invitrogen A32728 Dilute at 1:2k;
Rabbit polyclonal anti-NPM1 ABclonal A17983 Dilute at 1:1k;

Validation

All commercial antibodies have been validated by the manufacturers for applications, such as immunoblotting, immunofluorescence in human and mouse cells. The homemade anti-Tat and anti-TatR52ame2 antibodies were validated by dot blots or immunoblotting using synthetic peptides or lysates from 293T cells overexpressing indicated constructs or from E4 and/and 2D10 cell lines treated with or without TNFa. Results are shown in supplementary figure 5.

Eukaryotic cell lines

Policy information about cell lines and Sex and Gender in Research

Cell line source(s)

HEK293T, Hela and Jurkat T cells were obtained from ATCC. E4 and 2D10 cells were generously provided by J. Karn at Case Western Reserve University. Primary CD4+ T cells were isolated from healthy donors and HIV-1 patients on ART.

Authentication

Cell lines were obtained from and authenticated by ATCC. We indirectly verified their identity by their morphology, growth behavior or transcriptomic profiles. Primary CD4+T cells were authenticated by fluophore-labeled CD4+ antibody staining followed by flow cytometry analysis.

Mycoplasma contamination

All cell lines tested negative for Mycoplasma contamination

Commonly misidentified lines
(See ICLAC register)

This study does not use any commonly misidentified lines.

Flow Cytometry

Plots

Confirm that:

- [x] The axis labels state the marker and fluorochrome used (e.g. CD4-FITC).
- [x] The axis scales are clearly visible. Include numbers along axes only for bottom left plot of group (a 'group' is an analysis of identical markers).
- [x] All plots are contour plots with outliers or pseudocolor plots.
- [x] A numerical value for number of cells or percentage (with statistics) is provided.

Methodology

Sample preparation	Methods for the preparation of cells and their source were described in figure legends and Materials and Method section.
Instrument	Samples were analyzed using FACS Celesta flow cyotmeter.
Software	Flow cytometry data were collected on BD FACSDivaTm Software and analyzed using FlowJo v10.6.2.
Cell population abundance	At least 20,000 events were collected for each population analyzed in relevant figures.
Gating strategy	Using the FSC/SSC gating, debris was removed by gating on the main cell population. FSC-H/FSC-A were employed for gating singlets. DAPI negative cells were gated as viable cells and proceeded to gating for GFP+, GFP-, mKO2+ and mKO2- cells defined according to the appropriate untreated control cells.

- [x] Tick this box to confirm that a figure exemplifying the gating strategy is provided in the Supplementary Information.
